# The Cells of the Islets of Langerhans

**DOI:** 10.3390/jcm7030054

**Published:** 2018-03-12

**Authors:** Gabriela Da Silva Xavier

**Affiliations:** 1 Section of Functional Genomics and Cell Biology, Department of Medicine, Imperial College London, Hammersmith Hospital Campus, Du Cane Road, London W12 0NN, UK; g.dasilva-xavier@imperial.ac.uk or G.DaSilvaXavier@bham.ac.uk; Tel.: +44-(0)207-594-3358; 2 Institute of Metabolism and Systems Research (IMSR), University of Birmingham, Edgbaston B15 2TT, UK

**Keywords:** islets of Langerhans, insulin, glucagon, somatostatin, pancreatic polypeptide, ghrelin, pancreas, diabetes, endocrine

## Abstract

Islets of Langerhans are islands of endocrine cells scattered throughout the pancreas. A number of new studies have pointed to the potential for conversion of non-β islet cells in to insulin-producing β-cells to replenish β-cell mass as a means to treat diabetes. Understanding normal islet cell mass and function is important to help advance such treatment modalities: what should be the target islet/β-cell mass, does islet architecture matter to energy homeostasis, and what may happen if we lose a particular population of islet cells in favour of β-cells? These are all questions to which we will need answers for islet replacement therapy by transdifferentiation of non-β islet cells to be a reality in humans. We know a fair amount about the biology of β-cells but not quite as much about the other islet cell types. Until recently, we have not had a good grasp of islet mass and distribution in the human pancreas. In this review, we will look at current data on islet cells, focussing more on non-β cells, and on human pancreatic islet mass and distribution.

## 1. Introduction

The islands or (more commonly) islets of Langerhans, first described by their namesake- Paul Langerhans- in 1969, are islands of mixed populations of endocrine cells that are scattered in the parenchyma of the pancreas. Islets of Langerhans have been much studied in the context of diabetes due to the hormones produced and secreted from the cells which form these micro-organs, which are involved in the regulation of glucose homeostasis. The discovery of insulin, and the demonstration that it can lower blood glucose in dogs, by Frederick Banting and Charles Best in 1921, and its subsequent development for clinical use, in collaboration with John Macleod and James Collip, led to the award of the Nobel Prize in Physiology or Medicine to Banting and Macleod in 1923. Since then, the biology of the insulin producing β-cells in the islet came under much scrutiny as the loss of β-cell function, hence the loss of insulin, was associated with diabetes. Initially, much more associated with type 1 diabetes, which is characterised by the loss of β-cell mass due to autoimmune attack of this cell type, loss of functional β-cell mass is now also associated with type 2 diabetes (T2D).

There are multiple developmental cues for islet formation ranging from external cues such as nutritional status (i.e., the maternal nutritional state) to signals from the developing foetus e.g., from the central nervous system [1]. Replenishment of β-cell mass following the loss of significant β-cell mass (circa 90%) in mice has been shown to lead to activation of β-cell duplication [2,3]. Recuperation of β-cell mass following >90% loss of β-cell mass was shown to be via transdifferentiation of other islet cells, e.g., α-cells [4] and δ-cells [5], into β-cells. Data from the literature indicate that whilst loss of pancreatic β-cell mass may lead to disease, there is little or no effect on physiology from near complete loss of α-cell mass, responsible for secretion of glucagon (the counter hormone to insulin) in rodents [6]. With the advent of the possibility to switch islet cell identity as a means to alter islet cell mass as treatment for diabetes [4,5,7,8], it is perhaps timely to review current information on islet cell mass and the function of the different islet cell types.

## 2. Islet Distribution in the Pancreas: Structure, Size, Location

The total number of islets in a human pancreas has been estimated to be between 3.2 and 14.8 million, with a total islet volume of 0.5 to 2.0 cm^3^ [9,10,11,12,13,14]. The cellular composition and architecture of pancreatic islets differ between and within species [15,16]. The inter-species differences have previously been shown to correlate with functional differences [17]. Human islets consist of circa 30% glucagon-producing α-cells, circa 60% insulin producing β-cells, with the remainder circa 10% made up of δ-cells (somatostatin-producing), γ- or PP cells (pancreatic polypeptide-producing), and ε-cells (ghrelin-producing) [9,17,18,19,20], with these endocrine cells randomly distributed throughout the islet [15,16,17]. Rodent islets are widely used in biomedical research, and have a different architecture with a core of β-cells surrounded by the other endocrine cell types [16,21,22,23].

Recently, Ionescu–Tirgoviste and colleagues [9] performed a careful evaluation of islet characteristics from a healthy human pancreas by analysing pancreatic sections to produce a three-dimensional islet distribution map. The study revealed that the human pancreas contains on average 3.2 million islets, with a mean islet diameter of 108.92 μm (±6.27 μm), and a mean islet volume of 0.00069 μL (±0.00011 μL) [9]. The majority of islets (~66%) have a surface area of between 1000 and 10,000 μm^2^. Approximately 24% and 9% of islets had a surface area of more than 100,000 μm^2^ and less than 1000 μm^2^, respectively. The islets appear to have a threshold surface area of about 100,000 μm^2^, where “islets” that have apparently larger surface area than the threshold were made up of clusters of islets [9]. The smaller islets tended to cluster around blood vessels [9]. When islet surface area and distribution were averaged over pancreas section area, Ionescu-Tirgoviste and colleagues concluded that there was a uniform scattering of islets in the body of the pancreas, with the islets adopting an overall spherical structure [9]. This is in contrast to previous reports indicating an uneven distribution of islets in human [24] and rodent pancreas [21,25,26,27,28,29,30,31,32], although the differences may be due to differences in the methodologies used for estimating β-cell mass.

Islet mass and distribution in the pancreas may be an important consideration in light of the known metabolic consequences of pancreatectomy, where patients suffer a sudden loss of islet cell mass from surgical resection of the pancreas. Patients that have undergone a 50% (partial) pancreatectomy have reduced insulin secretion (circa 50%) but the region of pancreas that is removed was reported to have different metabolic impact [33]. Thus, removal of the pancreatic tail led to post-operative elevated fasting glucose and post-challenge glycaemia, whilst removal of the pancreatic head caused an improvement in oral glucose tolerance [33]. Earlier studies involving the loss of the pancreatic tail in human subjects also reported lowered insulin secretion and glucose tolerance in patients one year after partial pancreatectomy [34]. Intriguingly, the data from the pancreatectomy studies, together with Ionescu-Tirgoviste and colleagues’ report [9] that there was a uniform scattering of islets in the body of the pancreas, may imply that there are functional differences between the islets in the head and the tail of the pancreas. Thus, it would appear, not all islets are the same and there may be differences in the islet population in the head vs the tail of the pancreas. Such considerations are important beyond academic curiosity. The head and the tail of the pancreas have different developmental origins: the head of the pancreas is formed from the dorsal and ventral pancreatic bud, and the body and tail of the pancreas are formed from the ventral pancreatic bud [35]. Studies in rodents have previously shown that islets originating from the dorsal pancreatic bud had greater capacity to secrete and synthesise insulin than islets of ventral bud origins [26,27,28,29], perhaps hinting at potential programming differences during development in islets (reviewed extensively in [36]) in relation to their function and adaptive responses in adult life. This in turn may have an impact on the therapeutic strategies that may be pliable to increase functional β-cell mass in vivo (see Section 4 below) [26,27,28,29,33,35,36,37,38].

## 3. α-Cell

It would be fair to say that a lot less is known about the glucagon producing α-cell than the insulin producing β-cell. Glucagon is the counter hormone to insulin, and is typical of the fasted state. The concerted regulation of glucagon and insulin secretion is a major mechanism for the regulation of blood glucose. The major function of the counter-regulatory response is the prevention of hypoglycaemia, a response that is impaired in diabetes [37,38]. The importance of glucagon to the pathophysiology of T2D was first highlighted by Unger and colleagues in the bihormonal hypothesis which states that both hypoinsulinaemia and hyperglucagonaemia contributes to hyperglycaemia in T2D [39,40]. Thus, in T2D, α-cells display elevated glucagon secretion, enhanced glucagon secretion in response to amino acids, and ineffective suppression of glucagon secretion by high plasma glucose [41]. The importance of the regulation of α-cell function in the pathophysiology of T2D is currently the subject of intense research [42,43,44].

Glucagon secretion is regulated by both α-cell intrinsic and paracrine mechanisms. For example, it has been proposed that hyperglucagonaemia in T2D may be a result of the loss of intrinsic regulatory mechanism by glucose and amino acids, and extrinsic regulation by insulin and zinc, in the α-cells [41,45,46,47,48,49,50,51,52,53]. However, the loss of sensitivity to nutrients, insulin and zinc as mechanisms for hyperglucagonaemia have all been disputed [54,55,56,57,58]. The gut hormones—Glucagon-Like Peptide 1 (GLP-1) and Gastric Inhibitory Polypeptide (GIP)—have been proposed to be important in the regulation of glucagon secretion [59,60,61,62,63,64,65,66]. Additionally, there is evidence to suggest that GLP-1 can be produced in human islets, possibly in α-cells [67]. Somatostatin, secreted by pancreatic δ-cells, suppresses glucagon secretion [68,69,70]; it has been proposed that the inhibitory effect of GLP-1 on glucagon secretion [71] is effected indirectly via the stimulation of somatostatin secretion by GLP-1 [72].

The islets of Langerhans are innervated and subject to the regulation by the sympathetic and parasympathetic system such that glucagon secretion is stimulated in hypoglycaemia [73,74,75,76]. One characteristic of T2D is the progressive loss of the stimulation of glucagon and counter-regulatory hormones in patients treated with insulin [77,78]. Moreover, it has been reported that sympathetic innervation during pancreatic development is required for establishment of islet architecture and function [1], with the localisation of non-β cells a primary determinant of islet architecture [16,22,75,79]. Thus, the loss of non-β cell e.g. for the transdifferentiation into β-cells, may lead to the loss overall islet architecture, with implications on islet function.

In summary, the current evidence indicates that the intrinsic, nervous and paracrine regulation of glucagon secretion are not mutually exclusive, and likely act in concert [43,80].

**Figure 1:**
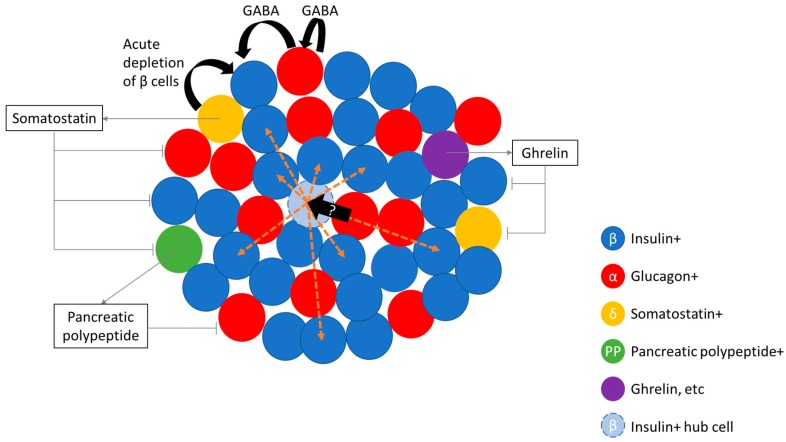
Schematic diagram showing the interdependency of islet cells. Evidence in the literature points to the possibility of the transdifferentiation (solid black arrows) of α-cells (red circle) via stimulation by gamma aminobutyric acid (GABA), and δ-cells (yellow circle) into insulin-containing β (like)-cells (blue circle). It is currently unclear whether the replenishment of β-cells from the transdifferentiation of α-cells is able to replace hub β-cells (light blue circle) which influence (yellow arrows) the function of other β-cells. Somatostatin, released from δ-cells, can inhibit the release of glucagon, insulin, and pancreatic polypeptide from α-, β-, and PP cells (green circle), respectively. Pancreatic polypeptide, released from PP cells, can inhibit the release of glucagon. Ghrelin, released from ghrelin-positive islet cells (purple circle), can inhibit insulin and somatostatin secretion.

Recently, Collombat and colleagues have demonstrated the potential to transdifferentiate endogenous α-cells in to β-like cells to replenish β-cell function through the administration of gamma aminobutyric acid (GABA) [7,8]. Furthermore, the authors showed that there was a continuous replenishment of glucagon-positive cells indicating that GABA treatment, not only induces transdifferentiation of α-cells in to β-like cells, but also maintains an α-cell pool [8]. This is important as it suggests that this treatment may be able to maintain the overall proportion of islet cell types (see Figure 1), although it is currently clear whether this will have effects on islet architecture and function in the long term. Nevertheless, this is a particularly intriguing finding as, whilst it has previously been demonstrated that near complete ablation of α-cells in rodents had little physiological effect [6], partial pancreatectomy in humans have been shown to lead to enhanced responsiveness to glucagon [81], and to secretion of glucagon from extra-pancreatic sites [82,83], both of which leads to altered glucose handling. Thus, although it is useful to be able to make β-like cells from α-cells, maintaining an α-cell population may also be important for the maintenance of glucose homeostasis in humans. 

In any cell (re)programming protocol, it is important to have a grasp of the identities/characteristics of the starting cell and the destination cell. The current state of the art is likely to act as a further catalyst for studies in to the α-cell as a result. For example, recent studies focussed on identifying β-cells based on expression profiles of protein-encoding genes and non-coding RNAs, are now also paralleled in α-cells [84,85,86,87,88,89,90,91,92,93,94,95,96,97,98,99,100,101,102,103,104,105].

## 4. β-Cell

The β-cell is the most studied of the islet cell types and has been written about copiously [106,107,108,109,110,111], so it would be superfluous to elaborate too much here. There are important differences between mouse and human β-cells which need to be considered in the design of therapeutic interventions for increasing functional β-cell mass [106]. 

What is a β-cell? Simplistically, it is a cell that makes insulin that is able to secrete insulin in response to a glucose challenge. Insulin is packaged into secretory granules at concentrations of circa 100 mM [112,113] as a complex with zinc, and released in response to high glucose (and other nutrient) concentrations [113] and stimulation by neurotransmitters that are released in response to food [114]. Insulin secretion is also enhanced by the presence of incretin hormones [114]. Somatostatin, which is secreted by neighbouring δ-cells, inhibit insulin secretion [115], as do epinephrine [116,117], galanin [118], ghrelin [119], leptin [120] and zinc ions [121]. Glucose metabolism in the β-cell differs from other cell types in that the presence of low affinity, high transport capacity glucose transporter(s) (GLUT1 and 2 in humans, Glut2 in mice) [122,123,124,125], and the low affinity hexokinase-glucokinase (GCK) [126,127] results in β-cell glucose metabolism being controlled by substrate availability. Glycolysis and mitchondrial oxidation are closely coupled, due to the lack of expression of “disallowed genes” such as lactate dehydrogenase (LDH) and the monocarboxylate transporter (MCT1)[128]. This coupling is disrupted in islets of Langerhans from type 2 diabetic donors [127,129,130], with concomitant expression of disallowed genes such as LDH and MCT1 (reviewed in [109]). Unsurprisingly there is currently much attention on the identity of the islet cell types, with the focus predominantly on the β-cell, especially in the context of coding and non-coding RNA (as mentioned in the Section 3), which may be involved in the regulation of the expression these disallowed or β-cell specific genes.

ATP production leads to closure of the ATP sensitive K^+^ channel (K_ATP_) channel on the cell surface; the subsequent membrane depolarization leads to opening of voltage-gated L-type calcium channels, influx of calcium and the release of insulin (detailed mechanisms reviewed in [106]). 

Interestingly, studies looking at β-cell regeneration in humans following partial pancreatectomy demonstrated an increase in β-cell function [131] but not β-cell mass [132]. Additionally, the same cohort of patients that had previously exhibited post-operative elevated fasting glucose and post-challenge glycaemia [33], showed amelioration of both parameters circa 3 years after pancreatectomy due to increased β-cell function [131]. Taken together with data from Ionescu-Tirgoviste and colleagues [9], which demonstrate that islet distribution is even throughout the human pancreas, the ability to increase function may not be dependent on the location of the β-cell in the pancreas. This compensatory increase in β-cell function is also seen in hyper-functional islets from obese individuals [113,133,134,135,136,137,138] which exhibit a two- to three-fold increase in insulin secretory capcity vs. lean individuals [139,140]. The obesity model also offers a contrary view to human β-cell mass plasticity *vs* pancreatectomy model: there is convincing data from various sources indicating that β-cell regeneration may occur in pancreases from obese subjects [135,140,141,142,143,144,145,146]. Add to this the potential to transdifferentiate α-cells to β-cells [8], and the potential of transplantation of human pluripotent stem cell-derived β-like cells [147], the future seems bright for restoring β-cell mass. However, one recent development may throw a spanner in the works. There is significant heterogeneity in β-cells within an islet which suggests that the structure of the islet—the connections between cells and the spatial arrangement of islet cells—may be important for the regulation of islet function [110,148,149]. Specialised insulin-positive islet hub cells—which are able to impact on the function of other cells in the islet—may be characteristically different to other insulin-positive cells in the same islet, begging the question as to how this difference was effected [110,148,149]. Did these cells arise from a special subset of progenitor cells, or were they the product of a subset of developmental cues that may have resulted from their position in the overall islet structure/pancreas? If that is the case then simply being able to (randomly) make more β-cells may not be enough to reinstate normal glucose homeostasis. For example, a question may be whether it is possible to replace hub β-cells through the transdifferentiation of α-cells (see Figure 1). It is worth mentioning that diabetes is increasingly acknowledged as a bihormonal disease and thus the study of at least both α- and β-cells are merited. It is possible that our view of the disease will expand as we find out more about the other islet cell types, e.g., the δ-cell (see below).

## 5. δ-Cell

Somatostatin secreting cells, or δ-cells, are present in the pancreatic islets, the hypothalamus, the central nervous system, peripheral neurons and the gastrointestinal tract [68,79,150,151,152]. δ-cells make up about 10% of the islet cell population. Somatostatin is a negative regulator of insulin, glucagon and pancreatic polypeptide secretion under conditions of nutrient stimulus [116,153,154,155,156], and in a Ca^2+^ dependent manner [156,157,158,159,160,161] (see Figure 1). δ-cells are electrically excitable, like α- and β-cells (reviewed in [111]). Ghrelin [162] and urocrotin [163] act on δ-cells leading to somatostatin release.Somatostatin is synthesised as a precursor molecule that is processed enzymatically by protein convertase, with the 14 amino acid peptide fragment, somatostatin (SST)-14, as the major peptide released by δ-cells [164,165]. SST-14 binds to the somatostatin receptor (of which there are five subtypes [166], which are G-protein coupled receptors, and lead to the inhibition of adenylyl cyclase [167] or activation of inwardly rectifying K+ channels [168].

δ-cells have been reported to transdifferentiate into β-cells in acute depletion of β-cell mass [5]. The transcription factor *Pax4* is involved in the development of both β- and δ-cells during pancreatic specification [169]. Recently it was shown that mis-expression *Pax4* in α-cells leads to conversion to β-like cells with no evidence for δ-cell like conversion [7]. It has been shown that the δ-cell fate is maintained by the *Hhex* gene [170]; loss of *Hhex* led to disrupted paracrine regulation of insulin secretion, which may potentially contribute to T2D [170,171]. Similarly, as δ-cells also regulate α-cells, loss of δ-cell mass due to transdifferentiation in to β-cells may lead to dysregulated glucagon secretion. In short, the current evidence does not support trasndifferentiation of δ-cells to β-cells as a viable means to replenish β-cell mass. 

## 6. PP Cell

Pancreatic polypeptide containing cells, also called PP cells or F-cells [172,173,174,175,176], make up 1–2% of the islet cell population [177,178,179]. PP cells are more concentrated in the head of the pancreas [180,181], where the cells are found to occupy the outer mantle of rodent islets or lining the capillaries in human islets [79]. Post-prandial pancreatic polypetide release is regulated by vagal and enteric nervous input [182,183,184,185], and is responsive to arginine but not glucose sitmulation [186]. Pancreatic polypeptide has been shown to be an inhibitor of glucagon release at low glucose [187] (see Figure 1). The major function of PP appears to be that of a satiety hormone (reviewed in [188]).

## 7. Ghrelin-Positive and Other Islet Cell Types

A further three types of islet cells have been described in the literature. These cells contain ghrelin [189,190], serotonin (enterochromaffin cells) [191,192], gastrin (G-cells) [174,193], and small granules of unknown content (P/D1- cells) [174,191,194,195,196,197].

Of these the ghrelin positive cells have recently attracted the most interest. Ghrelin-positive cells are mainly found in the gut [195,198,199,200,201]. Ghrelin-positive cells are also found in the islet, accounting for circa 10% and 1% of islet cell content in foetal and adult islets, respectively [119,189,190,195]. It has been suggested that ghrelin positive cells in the islets are in fact the P/D1 cells, which were described as containing an unknown hormone, as the two cell types share a lot of ultrastructural and distribution similarities [119]. The developmental programme for ghrelin positive cells appear to be different between human, mouse and rat (reviewed in [119]), and thus data on developmental pathways for ghrelin cells elucidated in mice (e.g., [202]) may not apply to humans.

Ghrelin is increased in fasting [203,204,205]; plasma ghrelin content has a reciprocal relationship with plasma insulin content [206,207], and has been shown to be an inhibitor of insulin secretion in human and rodents [208,209,210,211,212,213]. Ghrelin may also be a regulator of glucagon, PP and somatostatin release [210,214,215].

## 8. Conclusions

The cells in the islets have distinct regulatory function and operate within a complex regulatory network invoking paracrine and neuronal control of energy homeostasis. There are differences in islet architecture and distribution between human and experimental models, which may have an impact on islet function and energy homeostasis. Recent reports have indicated islet cells are plastic and that it may be possible to convert non-β islet cells into β-cells to replenish β-cell mass and function [4,5,7,8]. This knowledge may be important for the development of treatment strategies for diabetes, but will require careful evaluation of the impact of the loss of a particular non-β islet endocrine cell type on energy homeostasis.
